# Efficacy of vision therapy for unilateral refractive amblyopia in children aged 7–10 years

**DOI:** 10.1186/s12886-022-02246-9

**Published:** 2022-01-31

**Authors:** Yi-Ching Hsieh, Wen-Ling Liao, Yi-Yu Tsai, Hui-Ju Lin

**Affiliations:** 1 Department of Ophthalmology, Eye Center, China Medical University Hospital, No. 2, Yude Road, Taichung, Taiwan; 2 Graduate Institute of Integrated Medicine, China Medical University, Taichung, Taiwan; 3 Personal Medical Research Center, China Medical University Hospital, Taichung, Taiwan; 4 Department of Ophthalmology, China Medial University, Taichung, Taiwan; 5 School of Chinese Medicine, China Medial University, Taichung, Taiwan

**Keywords:** Amblyopia, Vision therapy, Part-time patching

## Abstract

**Background:**

There is a critical period for visual development, conventionally considered to be the first 6 years of life. Children aged 7 years and older are significantly less responsive to amblyopia treatment. This study investigated the efficacy of binocular vision therapy in amblyopic children aged 7–10 years.

**Methods:**

This retrospective study enrolled 36 children with unilateral amblyopia who were divided into a case group (receiving vision therapy, optical correction, and part-time patching of the weaker eye) and a control group (receiving optical correction and part-time patching of the weaker eye). Visual acuity (VA) was measured at baseline, at the 3-month, 6-month, and 9-month visits, and 3 months after cessation of treatment.

**Results:**

There were 19 subjects in the case group and 17 subjects in the control group. Mean VA in the case group improved from 0.39 ± 0.24 logMAR at baseline to 0.10 ± 0.23 logMAR at the endpoint of treatment (*p* < 0.001, paired t-test). Mean VA in the control group improved from 0.64 ± 0.30 logMAR at baseline to 0.52 ± 0.27 logMAR at the endpoint of treatment (*p* = 0.015, paired t-test). The improvement was significantly greater in the case group than in the control group (*p* = 0.006, two-samples independent t-test). All subjects underwent follow-up examinations within 6 to 12 months. There was no regression of VA in the case group 3 months after cessation of vision therapy. The patients in the case group who received visual therapy were with better VA improvement then patients with only optic correction and patching.

**Conclusions:**

Vision therapy combined with conventional treatment (optical correction and part-time patching) is more effective than conventional treatment alone in children aged 7–10 years with unilateral refractive amblyopia. The treatment results not only in greater vision gain, but also in shorter duration of treatment.

## Background

Amblyopia is the most common cause of monocular vision impairment in children, affecting 1 to 5% of the population worldwide [1, 2]. There are several known amblyogenic factors, such as strabismus, anisometropia, high refractive error, and deprivation from congenital cataract or congenital ptosis. Conventional treatments for unilateral amblyopia rely on depriving the fellow eye to force the use of the amblyopic eye, including patching and atropine penalization. However, 15 to 50% of amblyopic children fail to achieve normal visual acuity (VA) after extended periods of treatment [3–9]. Even when normal VA is achieved, amblyopia recurs in one-fourth of children [10, 11].

Currently, treatments for amblyopia are focused on the relationship between amblyopia and binocular vision, based on the hypothesis that binocular dysfunction plays an important role in amblyopia [12, 13]. For amblyopic children aged 7 years and older, who are less responsive to conventional treatments than younger children [14], the binocular approach is likely to yield a better outcome. Binocular amblyopic therapy includes any treatment in which both eyes are used, including dichoptic therapy, Interactive Binocular Treatment (I-BiT), dichoptic contrast balancing, binocular iPad game [15–17], and vision therapy [18].

Vision therapy is defined as an attempt to improve visual skills and abilities by optometrists initially. There are 2 main categories, including orthoptic vision therapy and behavior/perceptual vision therapy to improve binocular visual function [19]. Two review articles support the use of vision therapy for treatment of amblyopia [18, 20]. Hernández-Rodríguez demonstrated that vision therapy is a promising option for the treatment of children and teenagers with anisometropic amblyopia [20]. In adult amblyopia, perceptual vision therapy can enhance VA and visual performance [21, 22]. In this study, we evaluated the efficacy of vision therapy as treatment for amblyopia and compared this binocular approach with conventional treatments in amblyopic children aged 7–10 years.

## Methods

The study was a retrospective interventional comparative case series. The research protocol adhered to the tenets of the Declaration of Helsinki, and approval was obtained by China Medical University Hostpital Research Ethics Committee (CMUH110-REC2–002). All data were fully anonymized before access and analysis. Informed consent obtained from a parent or legal guardian as minor subjects were included in the study.

### Patient selection

The study enrolled amblyopic children from April 2016 to November 2019. The inclusion criteria were age 7 to 10 years; a diagnosis of unilateral amblyopia, with VA of 0.2 to 1.0 logMAR (20/32 to 20/200) with an interocular difference of at least 0.3 logMAR (≧3 lines); wearing glasses and part-time patching for ≧3 months before enrolment in the study to ensure that improvements in VA would be due to vision therapy rather than conventional treatment; stability of VA for 3 months; and follow-up for ≧6 months. The exclusion criteria were strabismus or other coexisting ocular disease, previous ocular surgery, previous vision therapy, ≧8 weeks premature birth, and developmental delay. Protocol.

In the case group, treatment consisted of vision therapy in the office for 1 h once a week for at least 3 months, in addition to wearing spectacles and receiving part-time patching from 4 to 6 h per day. In the control group, the children wore spectacles and received part-time patching 4 to 6 h per day only. Additional amblyopia training exercises at home, such as maze and connect the dots, for 30 min per day were suggested in both groups. Distance VA and cycloplegic refraction were measured at baseline, at the 3-month, 6-month, and 9-month visits, and 3 months after cessation of treatment. At the baseline visit, the eye examination included anterior segment examination, fundus examination, ocular motility, and cover test, in addition to distance VA and cycloplegic refraction. Patients with any type of strabismus and ocular diseases other than amblyopia were all excluded in this study, we ruled out the patients with binocular vision dysfunction as possible. Refractive error was measured by autorefractor Topcon RM-8900 before and after administration of cycloplegics, one drop of 1% mydriacyl (Alcon, Couvreur, Purus, Belgium), twice with a 10-min interval. Atropine is the gold standard for complete cycloplegia. Nevertheless, it takes 8–14 days to wash out, and it is not suitable for school-aged children in Taiwan due to the effect of blurred vision while reading and writing. The other drug used routinely for cycloplegic refraction is cyclopentolate; however, it is not available in our hospital. 1% Tropicamide is not as effective as cyclopentolate in inhibiting accommodation; nevertheless, it is a useful cycloplegic agent for measuring refractive error of myopia and hyperopia in children [23, 24]. Twenty minutes after the second drop of 1% Tropicamide, cycloplegic refraction was measured.

Distance VA was determined for each eye with the Landolt C chart at 5 m by one optometrist. Despite the fact that Landolt C chart does not have appropriate spacing like logMAR chart and does not come with crowding bar or forming a consistent crowding phenomenon, logMAR chart is inapplicable in Taiwan in which the language is not based on the Latin alphabet. Luminance values followed the recommendation for standard measurements of VA (160 to 200 cd m^− 2^). Stereoacuity was measured by the Stereo Butterfly Test (Stereo Optical Co., Inc.) according to the manufacturer’s instructions.

### Vision therapy equipment

Vision therapy includes orthoptic vision therapy and behavior/perceptual vision therapy (https://www.bernell.com/category/Vision-Therapy). Orthoptic training is composed of antisuppression charts, red-green bar reader, variable tranaglyphs, single and double Aperture Rule, Keystone Eccentric Circles, Lifesaver cards, Corian cheiroscope, Wolff standup cheiroscope, vectogram, mirror stereopscope, single oblique stereoscope, Keystone correct-eye scope, pencil push-ups, accommodative flippers, Brock string, and Hart chart. These tools can enhance vergence activity, accommodative activity, antisuppression training, and simultaneous vision. Combined with red-green goggles, Tranaglyphs can offer dichoptic presentation for binocular vision training. In addition, the Aperture Rule, cheiroscope, and stereoscope are designed for awareness of binocular vision and improvement of binocularity. Tranaglyphs, Aperture Rule, cheiroscopes, and stereoscopes are used to confirm binocular improvement. Take Aperture Rule for example, there are 12 cards with varying disparities. These cards are a combination of second-degree (flat fusion) and third-degree target (stereopsis). The patient will report double vision, suppression, or will be able to fuse the two targets and report one correct target. We can monitor the accuracy form the patients response and whether there is an improvement of binocularity. So as tranaglyphs and stereoscopes, these devices with feedback design are used to move patients to a slightly higher level by challenging them with different level of targets. Cheiroscopes serve a somewhat different purpose. The devices are used to improve binocular stability and reduce suppression. We will ask the patient to look into the cheiroscope, trace the target, and use a pencil drawing the target simultaneously. Binocularity is present if we observe the patient shifting their hands while attempting to trace, or actual drifts of drawing pictures. Suppression is noted if the patient reports parts of the picture disappearing.

Behavior training is composed of manual rotator, Marsden ball, Wayne saccadic fixator, Space fixator, and Eyeport vision training system. These tools can provide perceptual training, peripheral vision awareness, and eye–hand coordination exercises. Besides enhancing pursuit and saccade function, the program improves binocular function.

### Treatment program

In the one-hour vision therapy, not all of the subjects use exactly the same equipments; on the contrary, they received treatments followed the same rules. We divided the one-hour program into three parts. In the first quarter hour, the children were trained with equipments focused on the purpose of antisuppression, such as antisupression charts and Hart Chart. In the following half an hour, training depended on binocular vision skill was prescribed. The training tools with easier and higher acceptability, such as Keystone correct-eye scope, Corian cheiroscope, Wolff standup cheiroscope, vectogram and mirror stereopscope, were used in the first month. Variable tranaglyphs and Aperture Rule, with which the children were more difficult to achieve goal of fusion, were prescribed later according to the performance. In the last quarter hour, behavior training was given, because the tools were more interesting and funny for the children, who might be tired after training for forty-five minutes.

### Statistical analysis

Continuous data were presented as means and standard deviations, and categorical data were presented as proportions. We used *t*-tests to compare mean values of continuous variables and chi-squared tests to compare the frequencies of categorical variables between the two groups. Differences between the two groups in continuous variables were evaluated by two-samples independent *t*-tests, and differences in categorical variables were evaluated by chi-square tests. The paired *t-*test was used to compare the differences in VA between baseline and follow-up in each group. A *p*-value < 0.05 was considered to indicate a statistically significant difference. All statistical analyses were performed using SPSS Statistics 24 (IBM Corporation, Somers, NY, USA).

## Results

### Sample description

The case group included 19 children (7 girls and 12 boys) aged 7 to 10 years (mean, 7.78 ± 0.88 years). The control group included 17 children (10 girls and 7 boys) aged 7 to 10 years (mean, 8.31 ± 1.01 years). The baseline characteristics of the subjects are listed in Table 1. There were 18 anisometropic children in the case group and 13 anisometropic children in the control group. Isometropic patients were also included in the analysis of present study in spite of low proportion (13.9%) of unilateral amblyopia.. There were no significant differences between case and control group in all baseline characteristics. In the case group, the minimum and maximum hyperopic spherical equivalent (SE) was + 2.75 D and + 6.25 D, and the minimum and maximum myopic SE was − 0.50 D and − 19.25 D. Nevertheless, the patient with minimum myopic SE had high astigmatism (minus cylinder notation:+ 2.00–5.00 × 20). In the control group, the minimum and maximum hyperopic SE was + 1.00 and + 8.00 D, and the minimum and maximum myopic SE was − 1.25 D and − 18.75 D. Outcome analyses.

**Table 1:** Demographics and Baseline characteristics

	case (***N*** = 19)	control (***N*** = 17)	***P*** value
**Age**	7.78 (0.88)	8.31 (1.01)	0.109
**Sex**			0.187
**Male**	12 (63.2%)	7 (41.2%)	
**Female**	7 (36.8%)	10 (58.8%)	
**Anisometropia or Isometropia**			0.167
**A**	18 (94.7%)	13 (76.5%)	
**I**	1 (5.3%)	4 (23.5%)	
**Anisometropia definition met**			0.143
**Cylinder only (≥1.50D difference)**	1 (5.3%)	2 (11.8%)	
**Spherical equivalent only (≥0.50D difference)**	11 (57.9%)	10 (58.8%)	
**Spherical equivalent and cylinder**	7 (36.8%)	1 (5.9%)	
**Refractive error in amblyopia eye**			–
**0 to + 1.00D**	0 (0%)	1 (5.9%)	
**+ 1.00D to < + 2.00D**	0 (0%)	2 (11.8%)	
**+ 2.00D to < + 3.00D**	1 (5.3%)	1 (5.9%)	
**+ 3.00D to < + 4.00D**	1 (5.3%)	1 (5.9%)	
**+ 4.00D to < + 5.00D**	5 (26.3)	4 (23.5%)	
**≥ + 5.00D**	1 (5.3%)	4 (23.5%)	
**-1.00D to 0**	1 (5.3%)	0 (0%)	
**-2.00D to < −1.00D**	0 (0%)	2 (11.8%)	
**-3.00D to < −2.00D**	4 (21.1%)	0 (0%)	
**-4.00D to < −3.00D**	0 (0%)	0 (0%)	
**-5.00D to < −4.00D**	4 (21.1%)	1 (5.9%)	
**< −5.00D**	2 (10.5%)	1 (5.9%)	
**Depth of Amblyopia**			0.051
**severe (> 0.7 logMAR)**	2 (10.5%)	5 (29.4%)	
**moderate (0.3 to 0.7 logMAR)**	10 (52.7%)	12 (70.6%)	
**mild (< 0.3 logMAR)**	7 (36.8%)	1 (5.9%)	

Values are presented as N (%) or mean (SD)

*P* value for chi square test or two independent t test

*represent *P* value less than 0.05

**Fig. 1:**
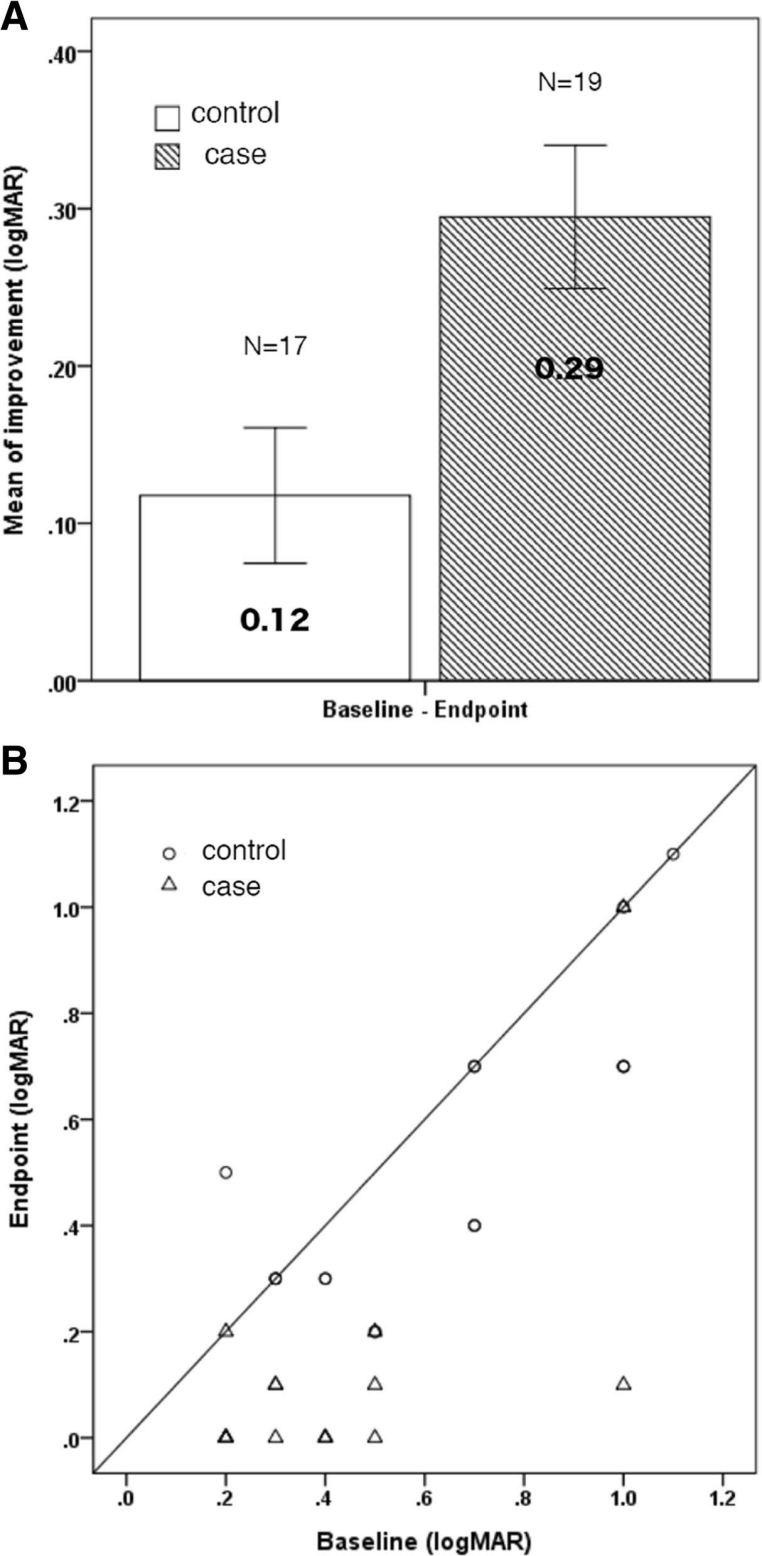
**A** Comparison of mean improvement in logMAR acuity in 2 groups. **B** Shown is BCVA of the amblyopic eye for the case (triangles) and the control (circles) group at endpoint visit. Data points below the line indicate improvement

At the endpoint visit, a significantly larger improvement in VA was found in the case group than in the control group, with a mean of 0.29 ± 0.20 logMAR (three lines improvement) versus 0.12 ± 0.18 logMAR (one line improvement) (*p* = 0.006, two-samples independent *t-*test). The average duration of treatment was 3.63 months (range, 3–9 months) in the case group and 4.41 months (range, 3–9 months) in the control group. In the first 3 months of treatment, the mean improvement in VA was 0.32 ± 0.20 logMAR in the case group (*n* = 18) versus 0.07 ± 0.18 logMAR in the control group (*n* = 13) (*p* = 0.005, two-samples independent *t-*test). At the 6-month visit, the mean improvement in VA was 0.23 ± 0.21 log MAR in the case group (*n* = 3) versus 0.12 ± 0.15 logMAR in the control group (*n* = 6) (*p* = 0.356, two-samples independent *t-*test). At the 9th month, the improvement in VA was 0.40 log MAR in the case group (*n* = 1) versus 0.30 logMAR in the control group (*n* = 1). One subject in the case group (5.26%) did not show improvement, versus eight subjects (47.06%) in the control group. Table 2 and Fig. 1A and B illustrate the improvement in logMAR acuity from baseline to endpoint visit in the two groups.

**Table 2:** Improvement of BCVA from baseline to follow-up visits in 2 groups

Variables	Δ^0–1^			Δ^0–2^			Δ^0–3^			Δ^0-E^		
	control	case	*P* value	control	case	*P* value	control	case	*P* value	control	case	*P* value
**logMAR**	*N* = 13	*N* = 18		*N* = 6	*N* = 3		*N* = 1	*N* = 1		*N* = 17	*N* = 19	
	0.07 (0.18)	0.32 (0.20)	0.005*	0.12 (0.15)	0.23 (0.21)	0.356	0.30 (−)	0.40 (−)	–	0.12 (0.18)	0.29 (0.20)	0.006*

Data presented as mean (SD). *p* value for two independent t test

Δ^0–1^: Difference between 3rd month and baseline

Δ^0–2^: Difference between 6th month and baseline

Δ^0–3^: Difference between 9th month and baseline

Δ^0–4^: Difference between 12th month and baseline

Δ^0-E^: Difference between endpoint and baseline

*represent *P* value less than 0.05

Table 3 shows the differences in VA between baseline and follow-up visits in both groups separately. In the first 3 months, the case group had a significant mean gain in logMAR acuity (*p* < 0.001, paired *t-*test), whereas there was no significant change in logMAR acuity in the control group (*p* = 0.147, paired *t-*test). At the 6-month visit, there were no significant changes in logMAR acuity in either the case or the control group (*p* = 0.192 and *p* = 0.110, paired *t-*test). At the endpoint visit, both groups showed significant improvement in logMAR acuity (*p* < 0.001 in the case group and *p* = 0.015 in the control group, paired *t-*test). In case group, mean acuity gain in severe amblyopia of was 0.45 ± 0.64 logMAR (*n* = 2), versus 0.35 ± 0.09 logMAR in moderate amblyopia (*n* = 10) and 0.17 ± 0.76 logMAR in mild amblyopia (*n* = 7). In control group, mean acuity gain in severe amblyopia was 0.18 ± 0.16 logMAR (*n* = 5), versus 0.13 ± 0.14 logMAR in moderate amblyopia (*n* = 12) and 0 logMAR in mild amblyopia (*n* = 1).

**Table 3:** Difference of logMAR acuity between follow-up visits and baseline

	Baseline	3rd month	*P* value	Baseline	6th month	*P* value	Baseline	9th month	*P* value	Baseline	Endpoint	*P* value
**control**												
**logMAR**	0.65 (0.30)	0.58 (0.27)	0.147	0.55 (0.31)	0.43 (0.33)	0.110	1.00 (−)	0.70 (−)	–	0.65 (0.30)	0.52 (0.27)	0.015*
**case**												
**logMAR**	0.39 (0.24)	0.07 (0.11)	< 0.001*	0.63 (0.32)	0.40 (0.53)	0.192	0.40 (0.00)	0.00(−)	–	0.39 (0.24)	0.10 (0.23)	< 0.001*

Data presented as mean (SD). *p* value for paired t test

* represent *P* value less than 0.05

**Fig. 2:**
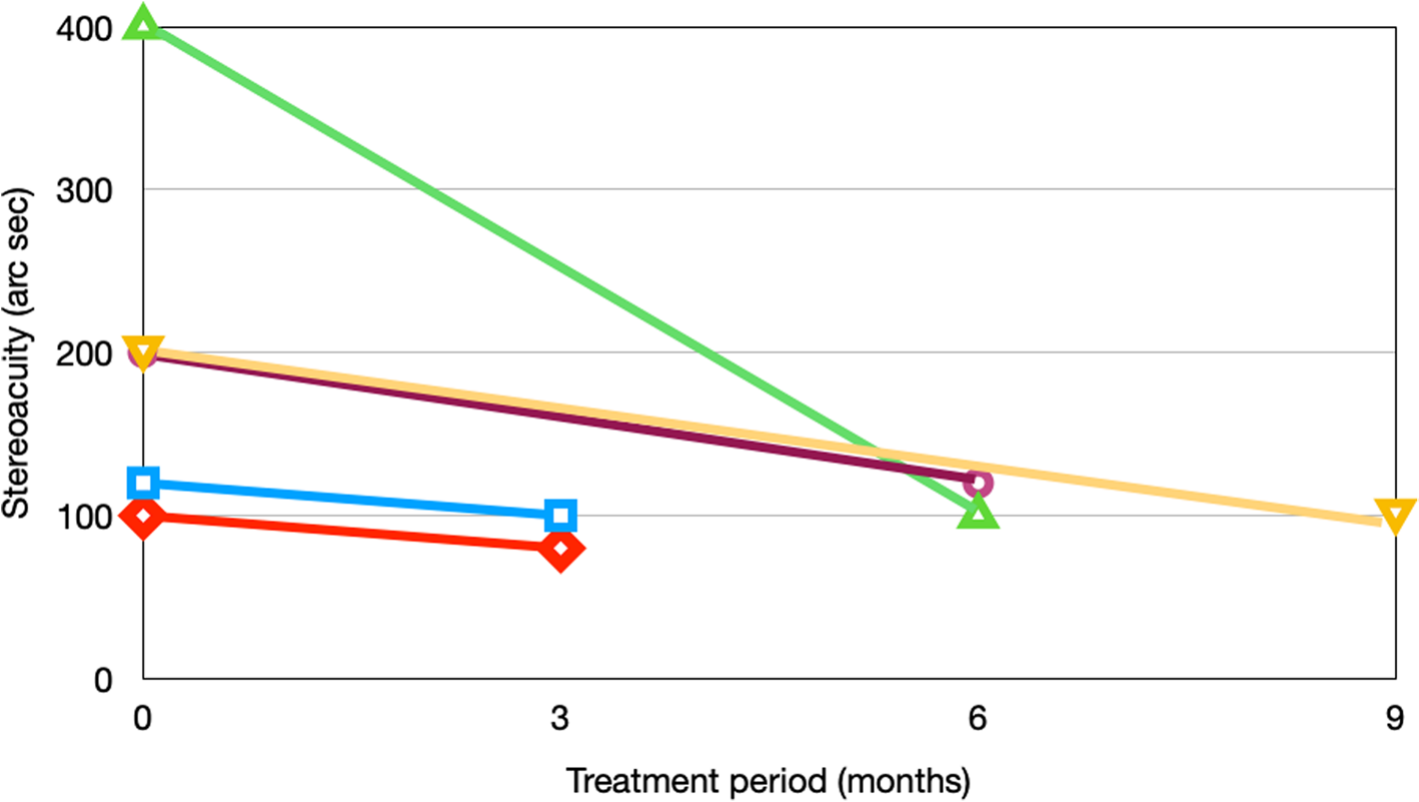
Stereoacuity for children who had complete measurement

In terms of stereoacuity, the complete data was limited to 5 subjects in the case group due to the nature of retrospective study. Mean stereoacuity was 200 s of arc (range 100–400). One subject showed no improvement. Overall, mean improvement of stereoacuity was 100 s of arc (range 0–300). (Fig. 2).

There were no adverse events, such as new tropia or diplopia, in the case group during the treatment period. There was no decline or further improvement in vision 3 months after treatment cessation.

## Discussion

Our study demonstrated that conventional treatment (optical correction combined with patching) is beneficial for unilateral amblyopia; however, vision therapy combined with conventional treatment resulted in significantly more improvement in VA in patients aged 7 years and older. The patients who included in case group were all received conventional therapy 3 months before they were attended case group, and they received visual therapy in addition to conventional therapy after they were enrolled in. We do the best to exclude the effect of visual therapy in advancement of VA was just occasional. First, there were no significant differences between case and control group in all baseline characteristics. Second, the range of follow-up duration was the same in both groups (3–9 months). Third, all subjects in the case group received the program with the same principles. The mechanism of the binocular approach played an important role in the improvement of VA in our patients.

Amblyopia, which was formerly considered a monocular disorder, is now considered to be a disorder resulting from binocular dysfunction. First, the risk of persistent amblyopia is elevated in abnormal stereoacuity. Bosworth showed that the risk of persistent amblyopia was 2.2 times higher among children 4 to 6 years old with nil stereoacuity [25]. In children with infantile esotropia, which is also a risk factor for abnormal stereoacuity, the risk of persistent amblyopia is elevated [26]. Second, it has been demonstrated that loss of binocularity is one of the features of amblyopia [27]. Finally, several neuroimaging studies suggest that depth perception from binocular disparity involves many cortical regions, and reduced responses in these areas are also detected in amblyopic patients [28]. Taken together, these studies support a positive association between binocular dysfunction and amblyopia.

The history of the binocular approach to amblyopia can be traced back to the 2000s. These approaches, including the I-BiT [15] system and “Push-Pull” [29], were aimed at the recovery of fusion and the re-establishment of binocular vision. In 2010, Hess et al. introduced dichoptic contrast balancing [16]. The goal of this approach was to restore binocular fusion and stereopsis and then strengthen the vision of the amblyopic eye. The authors used complementary dichoptic stimuli, which balanced the contrast between the amblyopic eye and the fellow eye, and the given visual task could only be completed by binocular integration. The study showed improvement in acuity of the amblyopic eye in adults. Later, the authors introduced an adaptation to provide dichoptic stimuli by iPad while playing the falling-blocks video game [30]. Other similar studies using binocular iPad games and viewing dichoptic movies by children also demonstrated positive results [17, 31].

A review article on binocular treatment of amblyopia concluded that there was no level I evidence to support the use of binocular treatment as a substitute for patching and optical correction [32]. Of importance, the present study emphasized vision therapy plus current treatment for amblyopia rather than replacement of current treatment. Furthermore, in addition to binocular dysfunction, there are deficits in contrast sensitivity, fixation, form and motion perception, saccades, and accommodation in amblyopic patients [33–36]. Vision therapy provides training not only for binocular vision, but also for visual skills and abilities, including accommodation and vergence, to improve visual processing. Some instruments also offer practice similar to perceptual learning, which was proven to improve VA in amblyopia [37, 38].

In the present study, vision therapy combined with optical correction and patching provided better results than conventional therapy in patients aged 7 years and older. In the case group, the patients had significant improvement in VA in the first 3 months (0.32 logMAR, *p* < 0.001, paired *t-*test), close to the endpoint results (0.29 logMAR, *p* < 0.001, paired *t-*test). The result suggests that at least 3 months of treatment would be suitable for most children 7 to 10 years old with unilateral amblyopia. Only one child showed no improvement even after 9 months of treatment. The difference in SE between the two eyes was–12.75 D. Nevertheless, another child, who had the second highest anisometropia (SE − 9.25 D), showed 0.2 logMAR improvement after 3 months of treatment. In patients with anisometropia more than − 10.0 D, vision therapy combined with patching and optical correction may be not as useful as in patients with SE less than − 10 D. To our knowledge, this is the first study to detect the limitation of vision therapy according to the severity of anisometropia. Regarding the relationship between severity of amblyopia and efficacy of vision therapy, the present study demonstrated most improvements of VA in severe amblyopia (0.45 ± 0.64 logMAR), followed by moderate amblyopia (0.35 ± 0.09 logMAR) and mild amblyopia (0.17 ± 0.76 logMAR). The results were similar to other literature that binocular therapy and behavioral training appear to be especially beneficial for more severe amblyopia [31, 39].

In the control group, there was no significant improvement in VA in the first 3 months, but there was improvement (0.13 logMAR) at the endpoint follow-up visit (average, 4.41 months) (*p* = 0.015, paired *t-*test). The result was consistent with other reports that occlusion therapy can improve VA, even after the age of 7 years [40, 41]. However, the improvement in VA was significantly larger in patients who received vision therapy (0.29 ± 0.20 versus 0.12 ± 0.18 logMAR; *p* = 0.006, two-samples independent *t-*test). Our result was similar to other reports that the binocular approach combined with patching provides a better result than patching alone [17, 42]. Moreover, with regard to the time required to get equivalent results, the binocular approach requires 10 to 20 h and patching requires 178 to 276 h to gain 0.2 logMAR improvement [8, 43, 44]. The time course of improvement is variable. In studies evaluating refractive correction for anisometropic amlyopia children, the range of improvement to best VA was 15 to 30 week [45, 46]. In our control group, it took an average of 4.41 months to achieve best VA, which was similar to other reports. In the case group, it took an average of 3.63 months to achieve best VA, and the most improvement occurred in the first 3 months. There are two possible reasons for the rapid improvement. First, vision therapy may provide stronger stimulation in visual processing compared to conventional treatment. Second, the improvement may be induced by strengthen and restore binocular interaction rather than treatments focusing in one eye.

In addition to the dose–response character of patching, another disadvantage of patching is lack of compliance. In the present study, the patients received vision therapy in the office under the optometrist’s instructions one on one, which provided ideal compliance with training. In contrast, compliance with patching is around 50% [47, 48]. In school-aged children, social and psychological withdrawal is another problem with patching. Besides vision therapy and patching, we encourage our patients undergo home training exercises 30 min a day in both groups. Despite frequent disruptions and the possibility of poor compliance, the sustained visual stimulation is required for treatment of amblyopia.

The efficacy of vision therapy for amblyopia is debated in current review articles and studies. Some author consider that there is insufficient evidence to recommend vision therapy [49, 50]. However, two review articles support the use of vision therapy for treatment of amblyopia [18, 20]. In the present study, we showed that vision therapy was effective for treatment of unilateral amblyopic children with a large proportion of anisometropia (94.7%). The result was consistent with the conclusion of Hernández-Rodríguez that vision therapy is a promising option for the treatment of children and teenagers with anisometropic amblyopia [20]. In adult amblyopia, perceptual vision therapy can enhance VA and visual performance [21, 22]. A possible mechanism is neural modification in the presence of brain plasticity [51, 52]. Larger randomized, controlled studies are needed to investigate the role of vision therapy in patients with different types of amblyopia and in different age groups.

In terms of stereoacuity, our data was limited due to the nature of retrospective study. There were 5 subjects with complete measurements of stereoacuity: at first visit and at the end of treatment. Following treatment, four of five children (80%) had improved stereoacuity. The studies involving binocular approach for amblyopia have reported that 0 to 86% achieved improved atereoacuity [17, 31, 53]. However, there was no evidence of improvements in stereoacuity among level I and II studies reported by Pineles et al. [27] The inconsistency may be due to the different stereoacuity tests employed, enrolled age, study design and types of amblyopia. Bossi M [31]. demonstrated that six of seven (85.7%) anisometropic amblyopia children had improved stereoacuity after binocular treatment, similar to our results. Children of refractive amblyopia may gain more improvements in stereoacuity from binocular approach than strabismic and deprivation amblyopia. Further randomized double-blind research is needed to determine the benefits of vision therapy in stereoacuity for different types of amblyopia.

One limitation of our study is the study design. This a retrospective study; therefore, the ability to control and randomize the subjects into case and control groups was limited. All subjects were included following the inclusion criteria after reviewing the charts. The statistical power was limited due to small sample size. According to the results we have, the improvement was significantly greater in the case group (*n* = 19) than in control group (*n* = 17), the study power was 66%. The statistical power will increase to 80% if there is 21 subjects in each group. However, we could not expand the subjects number due to the strict inclusion criteria and the nature of retrospective study design. In addition, the vision therapy program is not covered by health insurance in Taiwan. The family chose either vision therapy program or conventional treatment under the consideration of their budget. As a result, we could not assign subjects to case or control group randomly. The distribution of mild/moderate/severe amblyopia is not evenly distributed between the case and control group, although the *P*-value> 0.05. Besides subjects enrollment, the other main disadvantage of retrospective study is protocol design. Both objective and subjective outcomes measurements with conventional amblyopia workup should be performed, especially the evaluation of stereoacuity, which was incomplete in the current study. Further randomized controlled trials will be required to eliminate the disadvantages of retrospective study design. There are some limitations in the method. The way visual acuity and refraction performed is not the gold standard procedure. We use Landolt C chart because that LogMAR chart is inapplicable in Taiwan in which the language is not based on the Latin alphabet. Tumbling E version Bailey-Lovie Chart in lieu will be considered in the future for more precise visual acuity evaluation. When performing cycloplegic refraction, atropine is the gold standard drug. Nevertheless, it takes 8–14 days to wash out, and it is not suitable for school-aged children in Taiwan due to the effect of blurred vision while reading and writing. The other drug used routinely for cycloplegic refraction is cyclopentolate; however, it is not available in our hospital. Tropicamide is not as effective as cyclopentolate in inhibiting accommodation; nevertheless, it is a useful cycloplegic agent for measuring refractive error of myopia and hyperopia in children [23, 24]. Last, it is hard to standardize when it comes to conducting research using vision therapy. The first difficulty is the lack of precise criteria for placing a particular instrument in either category. Second, our subjects are children who are get tired and bored more frequently than adults. The scheduled training course might be interrupted unexpectedly. Moreover, vision therapy is similar to other types of therapy that involve learning and education. Motivation and interest influence the effect. Therefore, there are general principles mentioned in treatment program to maintain flexibility instead of hard and fast rules.

## Conclusions

Vision therapy combined with optical correction and patching is a more effective treatment than optical correction and patching alone in children from 7 to 10 years of age with unilateral amblyopia. The treatment results not only in greater vision gain but also in a shorter duration of treatment under the mechanism of binocular vision and perceptual learning. In patients with anisometropia greater than − 10.0 D, vision therapy combined with conventional treatment is less useful.

## Data Availability

All data generated or analysed during this study are included in this published article.
